# Lithium increases proliferation of hippocampal neural stem/progenitor cells and rescues irradiation-induced cell cycle arrest *in vitro*

**DOI:** 10.18632/oncotarget.5191

**Published:** 2015-09-08

**Authors:** Giulia Zanni, Elena Di Martino, Anna Omelyanenko, Michael Andäng, Ulla Delle, Kecke Elmroth, Klas Blomgren

**Affiliations:** ^1^ Center for Brain Repair and Rehabilitation, Sahlgrenska Academy, University of Gothenburg, Gothenburg, Sweden; ^2^ Karolinska Institute, Department of Women's and Children's Health, Stockholm, Sweden; ^3^ Karolinska Institute, Department of Physiology and Pharmacology, Stockholm, Sweden; ^4^ Department of Oncology, Institute of Clinical Sciences, Sahlgrenska Academy at University of Gothenburg, Gothenburg, Sweden; ^5^ Central European Institute of Technology, Masaryk University, Brno, Czech Republic

**Keywords:** lithium, hippocampus, radiotherapy, apoptosis, paediatric oncology

## Abstract

Radiotherapy in children causes debilitating cognitive decline, partly linked to impaired neurogenesis. Irradiation targets primarily cancer cells but also endogenous neural stem/progenitor cells (NSPCs) leading to cell death or cell cycle arrest. Here we evaluated the effects of lithium on proliferation, cell cycle and DNA damage after irradiation of young NSPCs *in vitro*.

NSPCs were treated with 1 or 3 mM LiCl and we investigated proliferation capacity (neurosphere volume and bromodeoxyuridine (BrdU) incorporation). Using flow cytometry, we analysed apoptosis (annexin V), cell cycle (propidium iodide) and DNA damage (γH2AX) after irradiation (3.5 Gy) of lithium-treated NSPCs.

Lithium increased BrdU incorporation and, dose-dependently, the number of cells in replicative phase as well as neurosphere growth. Irradiation induced cell cycle arrest in G_1_ and G_2_/M phases. Treatment with 3 mM LiCl was sufficient to increase NSPCs in S phase, boost neurosphere growth and reduce DNA damage. Lithium did not affect the levels of apoptosis, suggesting that it does not rescue NSPCs committed to apoptosis due to accumulated DNA damage.

Lithium is a very promising candidate for protection of the juvenile brain from radiotherapy and for its potential to thereby improve the quality of life for those children who survive their cancer.

## INTRODUCTION

The late-appearing neurocognitive decline observed in longitudinal follow-ups in children who receive cranial radiotherapy as tumour treatment remains a salient clinical issue and demands development of appropriate intervention strategies [1, 2]. The treatment-related sequelae encompass reduced memory formation capacity, attention deficits and a general reduction in ability to process information [3–5].

Irradiation of the juvenile brain is known to cause more severe damage in comparison to the adult brain. This may, at least partly, be explained by the higher neural stem/progenitor cell (NSPC) turnover in the young, differences in dynamic cell-autonomous regulation, as well as regional differences in growth [6–10]. NSPCs continuously repopulate the subgranular zone (SGZ) of the hippocampal dentate gyrus (DG) in the postnatal brain, displaying an age-dependent decline [11, 12]. Studies of hippocampal neurogenesis ablation using irradiation have shown that it plays a role in memory and information processing, providing a link between hippocampal neurogenesis and the decline of cognitive functions after radiotherapy [12–16]. Additionally, irradiation of the developing brain revealed that hippocampal neurogenesis was permanently halted and even displayed accelerated age-dependent decline [17], thus making this structure highly vulnerable. Compelling evidence supports the notion that irradiation-induced deregulation of the hippocampal NSPC cell cycle and growth leads to a further cascade of events in the neurogenic process that likely correlates with neurocognitive decline [18, 19]. In particular, the irradiation-induced long-term changes of the neurogenic niche are attributable to several factors, such as chronic inflammation secondary to increased apoptosis and sustained production of reactive-oxygen species (ROS) as well as direct DNA damage in NSPCs, which causes the cells to adopt a senescent phenotype and elevate cytokine secretion levels, ultimately resulting in increased glial differentiation *in vivo* [20–24]. Furthermore, the irradiation-induced effects on NSPC intrinsic properties include activation of the DNA damage response (DDR) due to the formation of DNA adducts, induced by single and double strand breaks, initially characterised by an increase in phosphorylated γH2AX, which may lead to cell cycle arrest through activation of cell cycle checkpoints kinases and ultimately apoptosis [25, 26].

Lately, increased awareness of the importance of hippocampal neurogenesis for memory function has prompted the development of pharmacological strategies aimed to protect this region during radiotherapy [27]. Nevertheless the radiosensitivity of NSPCs, even to low doses of radiation, still represents a major clinical concern and a suitable protective intervention is needed in order to prevent the neurocognitive alterations observed after radiotherapy [25, 28, 29].

Lithium is considered a promising drug in the treatment of radiotherapy-induced neurocognitive decline, as both *in vivo* and *in vitro* studies have shown that lithium has great potential for rescuing neurogenesis in the adult and juvenile brain after irradiation [30–34]. In addition, *in vitro* studies have proved lithium to be a specific radio-sensitiser for tumour cells [35] while rescuing adult neural stem and neuronal cell lines after irradiation, thereby increasing the therapeutic window such that it can be used in combination with radiotherapy [31, 34].

To our knowledge, the effects of lithium pre-treatment on hippocampal NSPCs from the juvenile brain in the context of irradiation have yet to be thoroughly examined and in this study we report our novel findings that lithium rescued *ex vivo* proliferation and cell cycle arrest of irradiated young hippocampal NSPCs. In agreement with previous studies, we found that lithium, applied as a pre-treatment and maintained after irradiation, moderately decreased DNA damage (γH2AX) and recruited a significant proportion of NSPCs into proliferation [31]. However, in contrast to previous reports we did not find any evidence of lithium preventing young NSPCs from irradiation-induced apoptosis, as judged by annexin V and Sub-G_1_ cell cycle analysis [34, 36].

## RESULTS

### Lithium has a concentration-dependent effect on NSPC proliferation

To investigate the effect of lithium on young NSPC proliferation, we used an *in vitro* neurosphere assay, which is a useful tool to investigate proliferation under diverse conditions and it is a valuable *in vitro* model system to study neurogenesis and neural development [37]. The isolated young NSPCs were grown in stem cell culture medium for 4 days until an average neurosphere diameter of 100 μm was reached.

**Figure 1:**
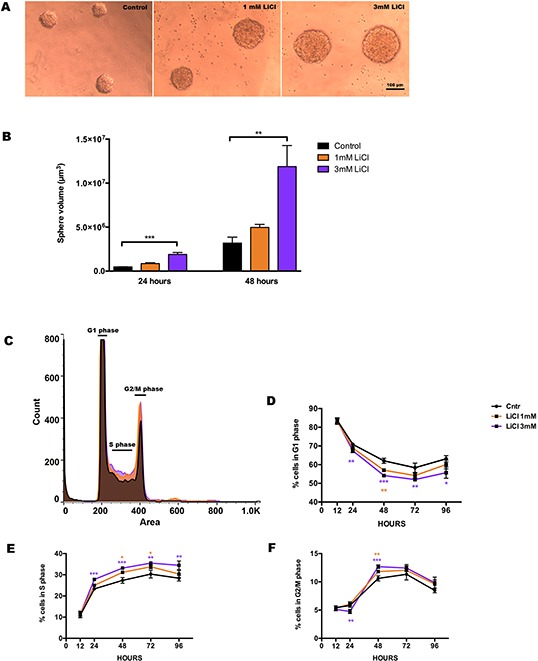
Lithium enhances neurosphere proliferation in a concentration- dependent manner **A.** Representative pictures of the neurospheres of neural stem/progenitor cells from the developing mouse hippocampus showing the dose response of lithium treatment on sphere size. **B.** The bar graph shows the quantification of the volume of the neurospheres in control (black), 1 mM LiCl (orange) and 3 mM (purple) at 24 hours *p_1 mM_ = 0.1830*, ***p_3 mM_ = 0.0001 and at 48 hours *p_1 mM_ = 0.8150*, **p_3 mM_ = 0.0043. **C.** Overlaid stacks of the propidium iodide (PI) histogram in control (black), 1 mM LiCl (orange) and 3 mM (purple) showing the relative distribution of the DNA content in: G_1_, S and G_2_/M. **D.** Quantification of the progressive reduction of cells in G_1_ phase at different times after the onset of lithium treatment. The effect started at 24 h for the highest dose of LiCl **p_3 mM_ = 0.0037 and it persisted at 48 h **p_1 mM_ = 0.0031, ***p_3 mM_ = 0.0001, at 72 h **p_3 mM_= 0.0075 and 96 hours *p_3 mM_ = 0.0271. **E.** Quantification of the percentage of cells in S phase at different times. At 24 h the effect of LiCl was significant at the highest dose ***p_3 mM_ = 0.0005 and it persisted at 48 h *p_1 mM_ = 0.0152, ***p_3 mM_ = 0.0010, at 72 h *p_1 mM_ = 0.0234 **p_3 mM_ = 0.0019 and 96 hours *p_3 mM_ = 0.0093. **F.** Quantification of the percentages of the cells in G_2_/M at different times showing the earliest effect at 24 hours at the highest does **p_3 mM_ = 0.0062, persisting at 48 hours *p_1 mM_ = 0.0224, ***p_3 mM_ = 0.0007. Data are presented as mean ± SEM, *n* = 3–6.

Lithium chloride (LiCl) was added post-dissociation to a single cell suspension and maintained until the analysis was performed, at 12, 24, 48, 72 and 96 hours. The neurosphere formation capacity reflects the proliferative potential and/or cell death of this cell type *in vitro* [38]. Therefore, we quantified the sphere volume at 2 time points, 24 and 48 hours (Fig. 1A), and we found that LiCl increased the volume of the clusters of dividing cells formed into neurospheres in a concentration- and time-dependent fashion (Fig. 1B). Control neurospheres had a mean volume (in μm^3^) of ≈ 0.49 × 10^6^, whereas neurospheres treated with LiCl had a mean volume of ≈ 0.85 × 10^6^ for 1 mM and ≈ 1.8 × 10^6^ for 3 mM LiCl after 24 hours exposure. After 48 hours we observed a similar response, with controls having a mean volume of ≈ 3.4 × 10^6^, whereas for 1 mM and 3 mM LiCl it was ≈ 4.9 × 10^6^ and ≈ 11 × 10^6^, respectively.

To confirm that this increase in neurosphere growth was due to LiCl acting on proliferation, we investigated the cell cycle distribution of the NSPCs using propidium iodide (PI) as a DNA label and performed univariate analysis of the DNA content in control, 1 mM and 3 mM LiCl-treated samples. (Fig. 1C). We found that lithium (3 mM) recruits up to 23% more cells to the S phase (Fig. 1E) already after 24 hours exposure, at the expense of a modest, 12%, reduction in the number of G_1_ phase cells (Fig. 1D), and a consequent increase of 19% in G_2_ phase cells (Fig. 1F) at 48 hours.

### Lithium protects young NSPCs from irradiation

**Figure 2:**
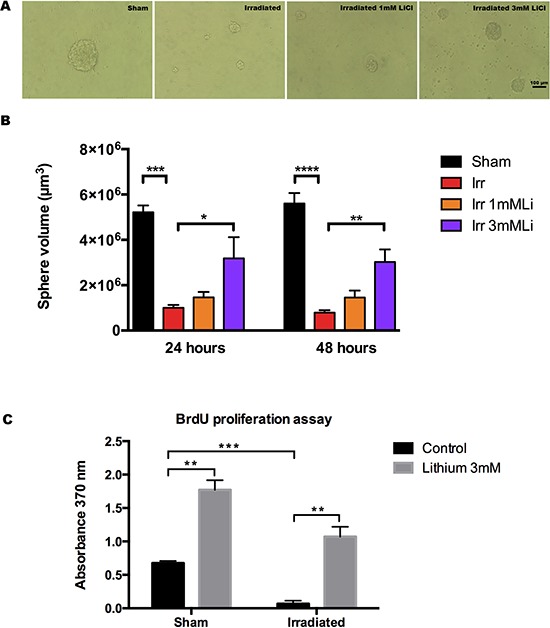
Lithium at 3 mM protects neurosphere proliferation after irradiation **A.** Representative pictures at 48 hours time after irradiation showing the neurosphere size in different conditions: sham, irradiated, irradiated+1 mM LiCl and irradiated+3 mM LiCl. **B.** Bar graph of the quantification of the volume of the neurospheres at 24 and 48 hours times after irradiation showing the effect of irradiation on reduction of neurosphere size at both times ***p_24 hours_ = 0.0002 and ****p_48 hours_ < 0.0001. The higher, but not the low, concentration of lithium significantly rescued the neurosphere size from irradiation damage *p_3 mM_24 hours_ = 0.0309 and **p_3 mM_48 hours_ = 0.0054. **C.** Bar graph of the quantification of BrdU 48 hours after irradiation showing that irradiation reduces significantly neural stem cells proliferation ***p_48 hours_ = 0.0004 and that 3 mM LiCl significantly increases neural stem proliferation both in sham and irradiated groups, respectively **p_sham_3 mM_ = 0.0012 and **p_irr_3 mM_ = 0.0021. Data are presented as mean ± SEM, *n* = 3–6.

To study the effect of different doses of LiCl after irradiation of NSPCs *in vitro* we used the neurosphere model and exposed a single cell suspension to a moderate irradiation dose of 3.5 Gy, as previously described [39]. NSPCs were pre-treated with 1 or 3 mM LiCl 12 hours prior to irradiation, maintained in LiCl-containing medium and assessed for neurosphere volume (Fig. 2A) at 24 and 48 hours after irradiation. This assay revealed that 3 mM lithium restored, earliest detectable at 24 hours, the growth capacity halted by irradiation (Fig. 2B). The 3.5 Gy irradiation dose caused a drastic reduction in growth that persisted even at the latest time point (72 h after irradiation). The mean sphere volume (in μm^3^) at 24 hours was ≈ 5.1 × 10^6^ in sham-irradiated NSPCs, whereas in irradiated NSPCs it was ≈ 1 × 10^6^. In the 3 mM lithium-treated, irradiated group the mean sphere volume was more than twice as big, ≈ 2.6 × 10^6^. Using 1 mM LiCl, however, we only observed a slight but not significant increase (≈1.3 × 10^6^). A similar scenario was observed 48 hours after irradiation (Fig. 2B), where we found that the mean sphere volume in sham-irradiated NSPCs was ≈ 5.5 × 10^6^ compared to ≈ 0.73 × 10^6^ in irradiated, ≈2.8 × 10^6^ in the 3 mM group and ≈ 1.4 × 10^6^ in the 1 mM group. To confirm the lithium effect on DNA synthesis (S phase), we pulse-labelled the cells with BrdU for 120 minutes, 48 hours after radiation exposure, with or without 3 mM LiCl. The BrdU incorporation assay (Fig. 2C) revealed that NSPC proliferation was increased by LiCl to a higher extent in the irradiated cells. BrdU incorporation was increased 2.6-fold in non-irradiated and 16-fold in irradiated NSPCs (Fig. 2C).

### Lithium recruits NSPCs into proliferation after irradiation-induced G_1_ arrest

**Figure 3:**
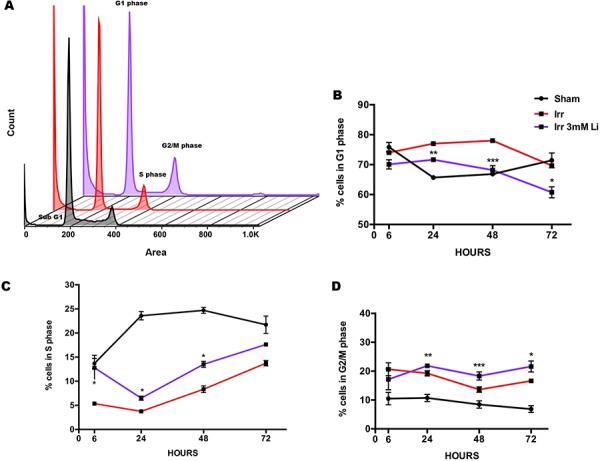
Lithium at 3 mM rescues proliferating cells in S phase **A.** Propidium iodide (PI) histograms representing the different phases of the cell cycle distribution in sham (black), irradiated (red) and irradiated+3 mM LiCl (purple). **B.** Quantification of the percentages of cells in G_1_ phase of the cell cycle providing additional evidence of irradiation inducing an arrest in this phase at 24 and 48 hours, ****p_24 hours_ < 0.0001, *p_48 hours_ = 0.0007. Lithium at 3 mM was able to reduce the proportion of cells arrested in G_1_ after irradiation at 24, 48 and 72 hours **p_3 mM_24 hours_ = 0.0070, ***p_3 mM_48 hours_ = 0.0006 and *p_3 mM_72 hours_ = 0.0121. **C.** Quantification over time of the proportion of cells in S phase shows that very few events were found in this phase after irradiation, indicating a negative effect on proliferation at any given time, **p_6 hours_ = 0.0012, ****p_24 hours_ < 0.0001, ****p_48 hours_ < 0.0001, **p_72 hours_ = 0.0018. Lithium at 3 mM dose restored the proliferating pool 6 hours after irradiation and this moderate effect was also observed at 24 and 48 hours, *p_3 mM_6 vhours_ = 0.0265, *p_3 mM_24 hours_ = 0.0359, *p_3 mM_48 hours_ = 0.0225. **D.** Quantification of the distribution of the cells in G_2_/M phase over time shows a pattern of accumulation in this cell phase after irradiation, **p_6 hours_ = 0.0074, ****p_24 hours_ < 0.0001, **p_48 hours_ = 0.0012, **p_72 hours_ = 0.0011. Even a more prominent stall in G_2_/M phase was observed after irradiation in combination with 3 mM LiCl treatment, **p_3 mM_24 hours_ = 0.0072, ***p_3 mM_48 hours_ = 0.0005, *p_3 mM_72 hours_ = 0.0232. Asterisks in the graph represent significant differences between the irradiated NSPCs and the irradiated NSPCs treated with lithium. Data are presented as mean ± SEM, *n* = 3–6.

To investigate the effect of LiCl on the cell cycle distribution of NSPCs after irradiation we performed cell cycle profile analysis. This analysis revealed that irradiation induced cell cycle arrest in G_1_, which is the cell cycle phase where most NSPCs were found following irradiation. Lithium at 1 mM had a mild effect in reducing this arrest in G_1_ at 24 and 72 hours (Supplementary Fig. 2B) but it did not influence the number of cells in S phase after irradiation (Supplementary Fig. 2C), and caused a prominent secondary arrest in the G_2_ phase 24 to 72 hours post-irradiation (Supplementary Fig. 2D). A similar scenario was observed for the higher dose of 3 mM at all time points from 6 to 72 hours after irradiation; however, we found that the G_1_-S phase transition was favoured, with a lower proportion of cells retained in G_1_ phase (Fig. 3B) and consequently higher numbers of cells in S (Fig. 3C) and G_2_/M phases (Fig. 3D) when compared to the irradiated non-treated group.

### Lithium did not reduce apoptosis of NSPCs after irradiation *in vitro*

**Figure 4:**
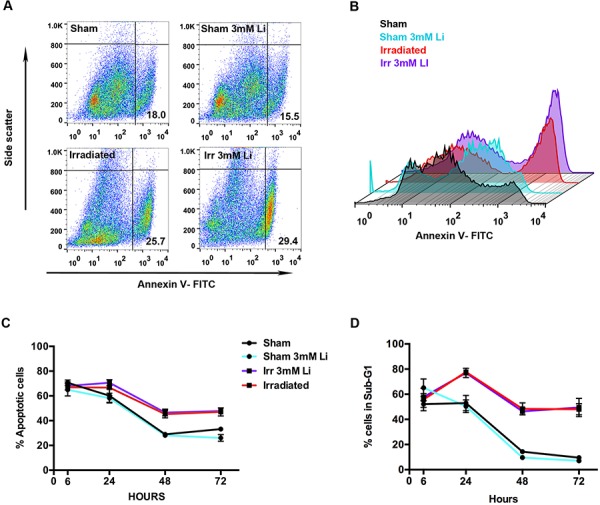
Lithium at 3 mM did not halt irradiation-induced apoptosis in young neural stem cells **A.** Representative scatter plot of the distribution of the cells stained with FITC for annexin V (x-axis) plotted against the side scatter (y-axis) analysed by flow cytometry showing the percentages (%) of apoptotic cell in the lower right quadrant in each group: sham, sham+3 mM LiCl, irradiated and irradiated+3 mM LiCl. **B.** Representative picture illustrating the peaks of intensity of annexin V-FITC (x-axis) plotted against the count of the events (y-axis) in sham (black), sham+3 mM LiCl (light blue), irradiated (red) and irradiated+3 mM LiCl (purple). **C.** Quantification of the percentage of apoptotic cells, positive for annexin V, at different times showing that irradiation strongly induces apoptosis in this cell type at 48 and 72 hours ***p_48 hours_ = 0.0004, ***p_72 hours_ = 0.0002. Lithium at 3 mM did not rescue this cell type from apoptosis. **D.** Time course of the percentage of cells in sub-G_1_. Irradiation displays an effect on cell death at 24, 48 and 72 hours, **p_24 hours_ = 0.0040, ****p_48 hours_ < 0.0001, ****p_72 hours_ < 0.0001. Lithium at 3 mM did not reduce the increased percentage of cells found in sub-G_1_. Data are presented as mean ± SEM, *n* = 3–6.

To examine whether LiCl also had an anti-apoptotic effect on NSPCs, annexin V and sub-G_1_ analyses were performed (Figs. 3A, 4A and 4B). The annexin V analysis showed that neither 1 mM (Supplementary Fig. 3C) nor 3 mM (Fig. 4C) LiCl treatment reduced the extent of irradiation-induced cell death. We observed a time-dependent increase in cell death due to irradiation up to 72 hours.

A similar effect was observed when analysing the sub-G_1_ fraction on the PI histogram (Supplementary Fig. 3D). This is a valid method to quantify cell death [40] and revealed a higher percentage of cellular debris 24 hours after irradiation (Supplementary Fig. 2D and Fig. 4D). However, lithium could not reduce or halt the committed cell death in our neurosphere model.

### LiCl reduced DNA damage after irradiation

**Figure 5:**
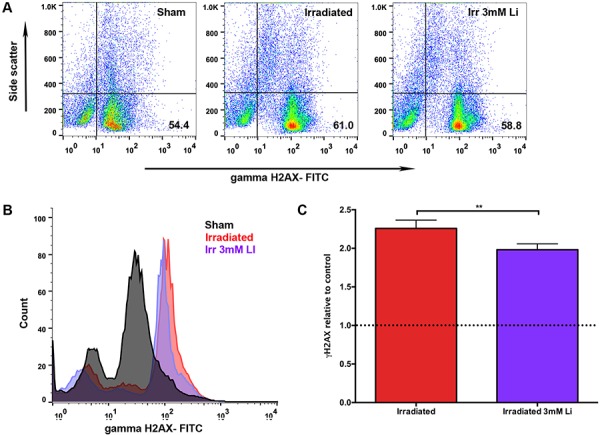
Lithium treatment reduced irradiation-induced DNA damage in young neural stem cells **A.** Representative scatter plot of the distribution of cells stained with FITC for γ-H2AX on the x-axis plotted against the side scatter on the y-axis showing the percentages (%) of DNA damage in the lower right quadrant in the sham, irradiated and irradiated+3 mM LiCl groups. **B.** Representative picture of the peaks of intensity for γ-H2AX FITC (x-axis) plotted against the count of the events (y-axis) in sham (black), irradiated (red) and irradiated+3 mM LiCl (purple). **C.** Bar graph illustrating the quantification of the mean peak intensity relative to sham control showing that irradiation significantly increased the double stranded break marker γ-H2AX, **p_irradiation_ = 0.0021 (red bar), whereas 3 mM LiCl was able to reduce this strongly induced marker of genotoxicity when compared to the irradiated group, **p_3 mM_irradiation_ = 0.0028 (purple bar). Data are presented as mean ± SEM, *n* = 3–6.

To investigate if lithium protected NSPCs from DNA damage induced by radiation, we quantified the levels of the DNA damage response marker γH2AX (Fig. 5A and 5B) 30 minutes after irradiation. Indeed, the normalised values showed that lithium reduced the radiation-induced DNA damage detected 30 minutes after irradiation. We found a 2.26-fold increase in phosphorylated γH2AX 30 minutes after irradiation whereas NSPCs treated with 3 mM LiCl displayed a 1.98-fold increase (Fig. 5C). Interestingly, this suggests that lithium maintains proliferation in NSPCs at least in part by attenuating DNA damage response.

## DISCUSSION

The aim of this study was to investigate the effects of different concentrations of LiCl on hippocampal NSPCs from the young, developing brain after irradiation *in vitro* in order to predict the response *in vivo*. Two relevant concentrations were chosen based on previous findings, including our own (Zanni et al., unpublished), showing that lithium at therapeutic doses (0.6–1.2 mmol/L) appears to accumulate in brain structures containing a higher proportion of cell bodies in general and in neurogenic regions in particular, suggesting that the brain concentrations may not reflect those in the blood [41, 42].

Lithium is the most potent mood stabiliser used in the treatment of bipolar disorders and it has proved to be useful also for several other diseases. Pre-clinical studies demonstrated that chronic lithium treatment protects against the neurodegenerative effects of cranial radiotherapy through its pro-neurogenic, anti-inflammatory and anti-apoptotic effects [30, 34]. Beneficial effects on rescued neurogenesis and synaptic plasticity in a Down syndrome mouse model have also been investigated [43] as well as other pre-clinical studies demonstrating the efficacy of lithium in preventing neural degeneration and restoring synaptic networks in diseases like Parkinson's disease, Alzheimer's disease and Fragile X syndrome [44, 45, 46]. There are as of yet limited published clinical data to support lithium as a neuroprotective or neuroregenerative agent. Hopes currently rest on future evidence to support the clinical use of lithium for the prevention of neurocognitive sequelae caused by cranial radiation therapy in children. The outcomes of current clinical trials in adults [47, 48, 49] will be valuable in the planning and safety assessment of paediatric trials. Valid concerns have been raised about lithium protecting not only neurons and neural stem cells, but also remaining tumour cells and thereby promote relapses [50, 51], even though these and other studies demonstrate that lithium does not promote tumour growth [34, 35, 50, 52]. Hence it is important to generate evidence supporting the applicability of lithium in childhood cancer patients.

As was previously described for adult hippocampal NSPCs, young NSPCs respond to lithium with increased proliferation and neurosphere formation *in vitro* in a concentration-dependent fashion (Fig. 1A and 1B) [32, 34]. Wexler et al. found that lithium acts on proliferation of adult hippocampal progenitor cells via inhibition of glycogen synthase kinase-3 beta (GSK3-β), thereby promoting DNA synthesis and proliferation through activation of the downstream Wnt-βcatenin signalling, and lithium in the range of 1–3 mM promoted neuronal differentiation [32]. We also tested the differentiation capacity of these cells and confirmed that lithium did not affect the lineage commitment of NSPCs (Supplementary Fig. 1A–1C). Additional previous studies observed that this increase in proliferative capacity of young NSPCs involved cell cycle entry and progress [52–56]. Higher proliferative potential of NSPCs seems to be correlated to shortening of the cell cycle [57–59] and indeed in the current study we found that the concentration-dependent effect involved re-distribution of NSPCs across the cell cycle. Accordingly, the percentage of cells in G_1_ phase was reduced (Fig. 1D) in favour of a marked increase in the S (Fig. 1E) and G_2_/M (Fig. 1F) phases. Taken together our findings from the cell cycle distribution suggest that lithium-treated NSPCs shortened the G_1_/S phase transition or the time in G_1_, resulting in increased proliferation as judged by both increased neurosphere growth and increased BrdU incorporation (Fig. 2C).

Next we investigated whether this lithium-dependent proliferative gain could rescue young NSPCs after irradiation, or rather promote radio-sensitization and apoptosis, as previously observed in cancer cell lines [35]. To address this question we exposed NSPCs to 3.5 Gy irradiation [60], resulting in a 5.3- and 7.5-fold decrease in neurosphere volume compared to sham at 24 and 48 hours after irradiation, respectively. The NSPCs receiving 12 hours lithium pre-treatment with 3 mM, but not 1 mM, displayed a 2-fold increase in neurosphere volume both 24 and 48 hours after irradiation (Fig. 2B). These data support previous studies where lithium was found to increase neurosphere growth *in vitro*, hence ruling out the possibility that this treatment may be sensitizing NSPCs in the developing brain to irradiation [36, 61]. Lithium has previously been observed to radio-sensitize cancerous cells and at the same time protect NSPCs, possibly due to lithium acting on genes with multifunctional roles in distinct DNA repair pathways, which are frequently aberrant in cancer [35, 62]. This doubly beneficial effect of lithium strongly argues in favour of concurrent lithium treatment during radiotherapy [31, 35, 62].

To further validate that the increased size of the irradiated neurospheres after lithium treatment was due to increased numbers of proliferating cells, rather than hypertrophy, we assessed BrdU incorporation in NSPCs 48 hours after irradiation (Fig. 2C), confirming that 3 mM LiCl recruits NSPCs into proliferation and restores the proliferative capacity halted after irradiation, as indicated by increased DNA synthesis also *in vivo* [30, 63]. In support of this, the cell cycle distribution of NSPCs after irradiation revealed that the reduction of NSPCs in S phase could be rescued by 3 mM, but not 1 mM, LiCl treatment, with full restoration of proliferation as early as 6 hours after irradiation, and maintained protection at least up to 72 h after irradiation (Fig. 3C and Supplementary Fig. 2C).

For regenerative purposes, manipulation of the cell cycle in proliferating cells seems to be a promising approach to therapy, and this is particularly relevant after irradiation in an attempt to restore the depleted pool of proliferating NSPCs [24, 58]. The DDR pathway is activated in response to irradiation-induced DNA damage, resulting in a cascade of events that ultimately promotes post-translational modification of proteins involved in DNA damage repair, modulation of apoptosis and/or cell cycle progression [26, 28]. In particular, actively proliferating cells make use of the cell cycle checkpoints to ensure there is enough time for repair to occur, guaranteeing faithful transmission of the genome to the daughter cells even after genotoxic stress [28]. It was previously observed that an arrest in the G_1_ phase following irradiation was related to the activation of p53, a tumour suppressor gene that in turn up-regulates p21 and p16^Ink4a^, cyclin-dependent kinase inhibitors, thus leading to cell cycle arrest [64–66]. Our cell cycle analysis revealed a 20% increase in G_1_ arrest of NSPCs 24 hours after irradiation, persisting for at least 72 hours, thus indicating that NSPCs respond to irradiation by halting the cell cycle progression, most likely in a p53-dependent manner [67, 68]. Interestingly, our results showed that lithium has a concentration-dependent ameliorating effect on the G_1_ arrest after irradiation, lasting at least up to 72 hours after irradiation (Fig. 3B and Supplementary Fig. 2B). Another interesting observation following cell cycle distribution analysis was the accumulation of NSPCs in G_2_ phase after irradiation at all time points post irradiation. This accumulation was even more prominent in lithium-pretreated irradiated NSPCs (Fig. 3D and Supplementary Fig. 2D). Our hypothesis is that at the time of irradiation there is a heterogeneous pool of NSPCs, with cells at different cell cycle stages, which activate different DDR signalling pathways. We propose that cells that have recently entered interphase are more likely to activate a p21-dependent G_1_ arrest, whereas the cells that were recruited into proliferation by lithium treatment and have initiated the elongation process, are instead prone to arrest in G_2_ phase [28, 69].

To gain further insight into the mechanism of action of lithium we tested the hypothesis that a higher proliferative capacity may be concurrent with a higher apoptotic rate as a form of homeostatic mechanism of self-renewal, as observed *in vivo* in the DG of the mouse brain [70, 71]. We investigated two parameters indicative of apoptosis/cell death: annexin V (Fig. 4A and 4B), which binds to the phosphatidyl serine (PS) expressed in early apoptotic cells, and the sub-G_1_ cell cycle fraction (Fig. 3A and Supplementary Fig. 2A), which reflects the population of dying cells with fragmented DNA [40, 72]. Our results revealed that irradiated NSPCs display, from 24 hours after irradiation onwards, a higher and sustained apoptotic rate, as judged by both annexin V and sub-G_1_ analysis at all time points analysed, and this increase in apoptosis was not reversed by lithium, neither in the irradiated nor in the sham NSPCs (Fig. 4C, 4D, Supplementary Fig. 3C and 3D). These results do not corroborate previous reports of lithium showing an anti-apoptotic effect after radiation injury [30, 34]. This discrepancy might stem from the fact that the study of Huo et al. was performed *in vivo* and the microenvironment as well as the vasculature play a pivotal role in modulating the apoptotic response of NSPCs to ionising radiation [73]; whereas in the study by Yazlovitskaya et al. they used the immortalised HT22 adult hippocampal cell line, which may display a different age-dependent radiosensitivity and different cell-autonomous factors compared to our *in vitro* neurosphere model of young primary NSPCs [8]. We propose that young NSPCs suffering DNA damage, which are generally driven into apoptosis [74], in the presence of lithium remain committed to their programmed cell death to the same extent as their untreated counterparts, and therefore our conclusion is that lithium prevents the potentially carcinogenic transmission of damaged NSPCs bearing accumulated genotoxic stress through cell division.

As others and we have demonstrated that LiCl has a pro-proliferative effect, it may be argued that the apparent protection is merely a result of increased numbers of unaffected NSPCs rather than a specific protection against the effect of radiation. To further dissect the mechanisms of lithium-mediated neuroprotection after irradiation we sought to address the effect of LiCl on the DNA damage response of NSPCs after irradiation. Protection mechanisms include activation of the DNA-dependent protein kinase (DNA-PK) that in turn modulates the pro-survival PI3K/Akt pathway causing a decrease in γH2AX foci and an increase of the non-homologous end joining repair (NHEJ) pathway [31, 34, 75, 76]. In line with this, we found a reduction in radiation-induced γH2AX activation in 3 mM LiCl-treated NSPCs (Fig. 5C), indicating that this concentration of lithium was sufficient to produce a significant, although modest, decrease in the irradiation-induced DNA damage response, providing supporting evidence that the lithium mediated-rescue in proliferation of NSPCs is also accompanied by less genotoxic stress response and possibly a higher degree of protection.

In summary, the results from our *in vitro* model of hippocampal neurogenesis in the juvenile brain showed that NSPCs are protected by lithium, as reflected by higher proliferation rates without a reduction in apoptosis. The increase in proliferation is due to a shortening of the cell cycle, as shown by increased percentage of cells in S phase, and is accompanied by a reduction in irradiation-induced DNA damage. These findings strongly encourage future clinical studies of lithium in young patients receiving cranial radiotherapy.

## MATERIALS AND METHODS

### Animals and ethical permission

For all *in vitro* experiments hippocampal NSPCs were prepared from postnatal day 8 female C57BL/6J mice obtained from Charles Rivers Laboratories (Sulzfeld, Germany). Animals were delivered with their respective dams, housed under standard conditions of daylight (12-hour light cycle) and provided food and water *ad libitum* at the animal facility in Gothenburg (Laboratory of Experimental Biomedicine, EBM). All experimental conditions were approved by the Gothenburg Animal Research Ethics Committee, in accordance with the national animal welfare legislation. The following ethical identification number was used: 20-2013.

### Neural stem cell isolation and culture

Postnatal day 8 C57BL/6J mice were used for all *in vitro* experiments. The dams were euthanised after the pups were sacrificed. Each litter accounts for *n* = 1 and each time point has an n of 3 or 6.

Mice were decapitated without prior exposure to anaesthesia. Decapitation without anaesthesia is particularly rapid in these very young mice and minimises post-mortem changes in neural tissues [77]. Euthanasia solely by decapitation is considered an acceptable and preferable method for mouse pups [78].

The brains were rapidly removed and kept in HibernateA solution (Gibco/Invitrogen, United Kingdom). The hippocampi from each litter (12 hippocampi in each group) were pooled in the same tube and stored in ice cold HibernateA (Gibco/Invitrogen, United Kingdom).

The tissue was chopped and digested for a total of 20 minutes at 37°C in 0.01% papain (Worthington/Cell Systems, United States), 0.1% Dispase II (Roche, Indianapolis, United States), 0.01% DNAse I (Worthington/Cell Systems, United States), 12.4 mM MgSO_4_ in HBSS without Ca^2+^ and Mg^2+^ (Hank's Balanced Salt Solution, Worthington/Cell Systems, United States). The homogenate was spun down at 500 × g for 5 minutes and re-suspended in 1 ml new medium consisting of NeurobasalA (Gibco/Invitrogen, United Kingdom) with addition of 1X Glutamax (Gibco/Invitrogen, United Kingdom), 1X B27 without vitamin A (B27 w/o vitamin A, Gibco/Invitrogen, United Kingdom), penicillin (100 U/mL, Gibco/Invitrogen, United Kingdom) and streptomycin (100 μg/mL, Gibco/Invitrogen, United Kingdom). Here we used B27 w/o vitamin A, because the latter can be converted into retinoic acid, which causes the NSPCs to differentiate [79, 80], and our purpose was to keep the NSPCs in their most undifferentiated state.

Viable cells were counted using a Burker chamber and seeded at a concentration of 3 × 10^5^ cells/5 mL of culture medium in a T25 flask. A proliferating state was maintained by addition of fibroblast growth factor 2 (FGF-2, 20 ng/ml, PeproTech, United States of America), epidermal growth factor (EGF, 10 ng/ml, Gibco, United States of America) and heparin (10 μg/ml, Sigma-Aldrich, United Kingdom).

Every 4^th^ day, when the neurospheres reached an inhibiting size, they were passaged. The inhibiting density is reached when the diameter of the neurospheres is nearly 100 μm [81] and the centre of the sphere displays a dark core. These features were analysed under a brightfield microscope (Nikon TMS-F). For passaging, cells were collected by centrifugation and a single cell suspension was obtained by mechanical disruption of the spheres using a 1000-μl pipette as well as addition of 500 μl of TrypLE (trypsin, Gibco/Invitrogen, United Kingdom). PBS without Ca^2+^ and Mg^2+^ (PBS w/o Ca^2+^ and Mg^2+^, Gibco/Invitrogen, United Kingdom) was used to rinse the cells after trypsin treatment. Cells were fed growth factors every 2^nd^ day and cultured at 37°C in 5% CO_2_ and 95% air with 100% humidity. The analysis was performed after four passages to allow for a homogeneous selection of the neural stem cell population, thus keeping the genetic background of origin, avoiding chromosomal aberration [82, 83].

### *In vitro* lithium administration

Lithium chloride, LiCl (Sigma Aldrich, St. Louis, USA), was added to the single cell suspension at the 4^th^ passage at 2 different concentrations: 1 mM or 3 mM [32]. Analyses were performed at 12, 24, 48, 72 and 96 hours after the last passage. Between the last passage and analysis the cells were kept under proliferating conditions, as mentioned above, and fed 48 hours after the last passage. For the irradiation experiments, cells were pre-treated 12 hours prior to irradiation with 1 or 3 mM LiCl after the last passage and the analysis was performed 6, 24, 48 or 72 hours after irradiation.

### *In vitro* irradiation

A photon ^60^Co irradiation source (TEM Mobaltron Therapy Unit, Crawley, UK) was used to expose the NSPCs in single cell suspension at a set distance of 0.8 m and an absorbed dose of 3.5 Gy. Cells were irradiated at 1.3 × 10^6^ cells/ml density at 37°C for cell cycle and annexin V analysis, whereas for γH2AX cells were kept at 2°C to prevent DNA repair during the irradiation procedure. After irradiation, cells were placed back in their respective culture medium and kept in the incubator until flow cytometry analysis was performed.

### Cell cycle, annexin V and γH2AX flow cytometry analysis

At the time of analysis a single cell suspension was obtained as described above, except for replacing TryplE with 0.02% EDTA (Titriplex^®^ III. Z.A, E. Merck D-6100, Darmstadt). EDTA was used to preserve cell viability and to avoid annexin V leakage over the cell membrane due to trypsin treatment. For the entire analysis cell suspensions were triturated through a syringe and filtered through a nylon filter (pore size 41 μm, Millipore) to dispose of possible aggregates.

The propidium iodide (PI) staining solution consisted of 50 μg/ml PI (Molecular Probes^®^, Eugene, Oregon, United States), 0.1 mg/ml RNase A (Pure link™ Invitrogen, United States) and 0.6% Tergitol-type NP-40 (Sigma Aldrich, United Kingdom). DNA content frequency histogram was obtained using CellQuest Pro™ software (BD Biosciences, Stockholm, Sweden), where cell counting was plotted on the y-axis and PI fluorescence pulse area (FL2-A) on the x-axis. Univariate analysis was performed using ModFit LT V 3.0 (Verity Software house, Inc. Topsham, ME, USA).

Quantification of apoptosis was performed using the Alexa Fluor^®^ 488 AnnexinV cell apoptosis kit (Molecular Probes^®^) as per manufacturer's instructions at a concentration of 0.2 μg/ 1 × 10^6^ cells/ml. We did not use concomitant labelling of annexin V with PI because laser compensation was not possible for this cell type, resulting in all events being consistently double-positive for both markers.

Quantification of DNA double strand break repair was detected 30 minutes after radiation by washing the cells in PBS and putting them in blocking buffer containing FITC-conjugated anti-γH2AX antibody (#16–202A, Millipore, Billerica, USA) for 3 hours and washed again in PBS prior to flow cytometry analysis.

For both annexin V and γH2AX the signal intensity of irradiated samples was correlated to the signal from non-irradiated cells for each condition and repair time as previously reported [84].

PI, annexin V and γH2AX analyses were performed using a flow cytometer (BD FACS Calibur ™) with a 488 nm argon laser.

NSPCs were detected by granularity and cell size by using side scatter (SSC) and forward scatter (FSC) of the flow cytometer.

### Sphere size quantification (proliferation assay)

After the last 4^th^ passage single cells were plated at a density of 3 × 10^4^ cells/ml or after irradiation at a density of 5 × 10^4^ cells/ml in an uncoated μ-Slide 8 well (iBidi, GmbH, Martinsried, Germany). Representative pictures for each n were taken in 4 consistent regions of each well using a Leica DFC 295 camera and a Nikon TMS-F light microscope. For the irradiation experiment a Nikon ECLIPSE TE20 microscope with an INFINITY1–2C camera and Infinite analyze software were used to capture the pictures.

The diameter of each sphere was measured and the corresponding volume (in μm^3^) of the sphere was calculated offline for the 24 and 48 hour time using Image J 1.45s (Wayne Rasband, NIH, USA).

### Proliferation assay

Proliferation was evaluated using a 5′-Bromo-2′Deoxyuridine (BrdU) cell proliferation kit (Roche, Manheim, Germany). After irradiation cells were kept in T75 flasks in culture medium, with growth factors, for 48 hours. Cells were pulse-labelled with 10 μM BrdU for 120 minutes and then plated in a flat-bottom 96-well culture plate coated with poly-L-ornithine/laminin at a density of 5 × 10^3^. Cells were allowed to attach for 24 hours and the assay was then performed as recommended by the manufacturer.

### Differentiation

Differentiation of NSPCs was performed to demonstrate that they were multipotent and to investigate if lithium had any effect on lineage commitment. At the 5th passage they were plated as single cell in 19 mm Ø glasses, coated with 100 μg/ml polyornithine (Sigma-Aldrich, Stockholm, Sweden) and 50 μg/ml laminin (Gibco/Invitrogen, United Kingdom) in a 12-well plate. In each well the seeding density was 6 × 10^4^ NSPCs in 2 ml medium. The differentiation assay consisted of a gradual withdrawal of the growth factors in order to minimise cell death. The first 2 days the NSPCs received 10 ng/ml EGF and 10 ng/ml FGF, followed by 1 day of 5 ng/ml FGF and 4 days of complete withdrawal. Every second day half the medium was replaced. After 7 days in culture, the medium was removed, the cells were washed in PBS and fixed in 4% paraformaldehyde, PFA (Merck-VWR, Stockholm, Sweden) for 20 minutes at 4°C. Cells were washed twice in PBS for 10 minutes and put in blocking solution (0.2% Triton X-100, 5% donkey serum (Jackson ImmunoResearch Laboratories Inc., Cambridgeshire, UK), PBS) for 1 hour at room temperature on a shaking plate. Primary antibodies were incubated for 24 hours at 4°C on a shaking plate, and secondary antibodies for 2 hours at room temperature on a shaking plate. Both steps were performed in the blocking solution mentioned above. Between primary and secondary antibodies, cells were washed twice in PBS for 10 minutes. The following primary antibodies were used separately: mouse polyclonal anti-GFAP (1:500 Merck Millipore, Billerica, USA) and mouse monoclonal anti-MAP2 (2a-2b) (1:500 Sigma- Aldrich, Saint Louis, Missouri, USA). All secondary antibodies were used at 1:1,000 dilution and the following antibodies were used: Alexa Fluor^®^ 488 and 555 Donkey anti-mouse IgG (H+L). Cells were coverslipped with ProLong Gold containing 4,6-diamidino-2-phenylindole (Molecular Probes Inc., Eugene, OR, USA) for nuclear counter staining. Cells were counted as a percentage of positive cells for each lineage marker over the number of DAPI-positive cells using a semiautomatic stereology system (Stereo Investigator, MicroBrightField Inc; Colchester, VT, USA) under fluorescent light.

### Statistical analysis

Statistical analysis was performed using GraphPad Prism^®^ (La Jolla, CA, USA). All data are expressed as mean ± standard error of the mean (SEM). Statistical differences presented here were calculated using either a 2-way ANOVA when 2 treatments, irradiation and lithium, were compared or a one-way ANOVA for one treatment, only lithium, comparison. These tests of variances were followed by a Bonferroni post-hoc test for multiple comparison correction. A two-tailed unpaired *t*-test was used for the lineage commitment analysis. *P < 0.05* was considered statistically significant.

## SUPPLEMENTARY FIGURES


