# Supplementary material for: Lithium increases proliferation of hippocampal neural stem/progenitor cells and rescues irradiation-induced cell cycle arrest in vitro
Source: Oncotarget. 2015 Sep 8;6(35):37083–97. doi: 10.18632/oncotarget.5191 (PMC4741917; doi:10.18632/oncotarget.5191)
Supplement: Supplementary file 1 [file oncotarget-06-37083-s001.pdf]

SUPPLEMENTARY FIGURES

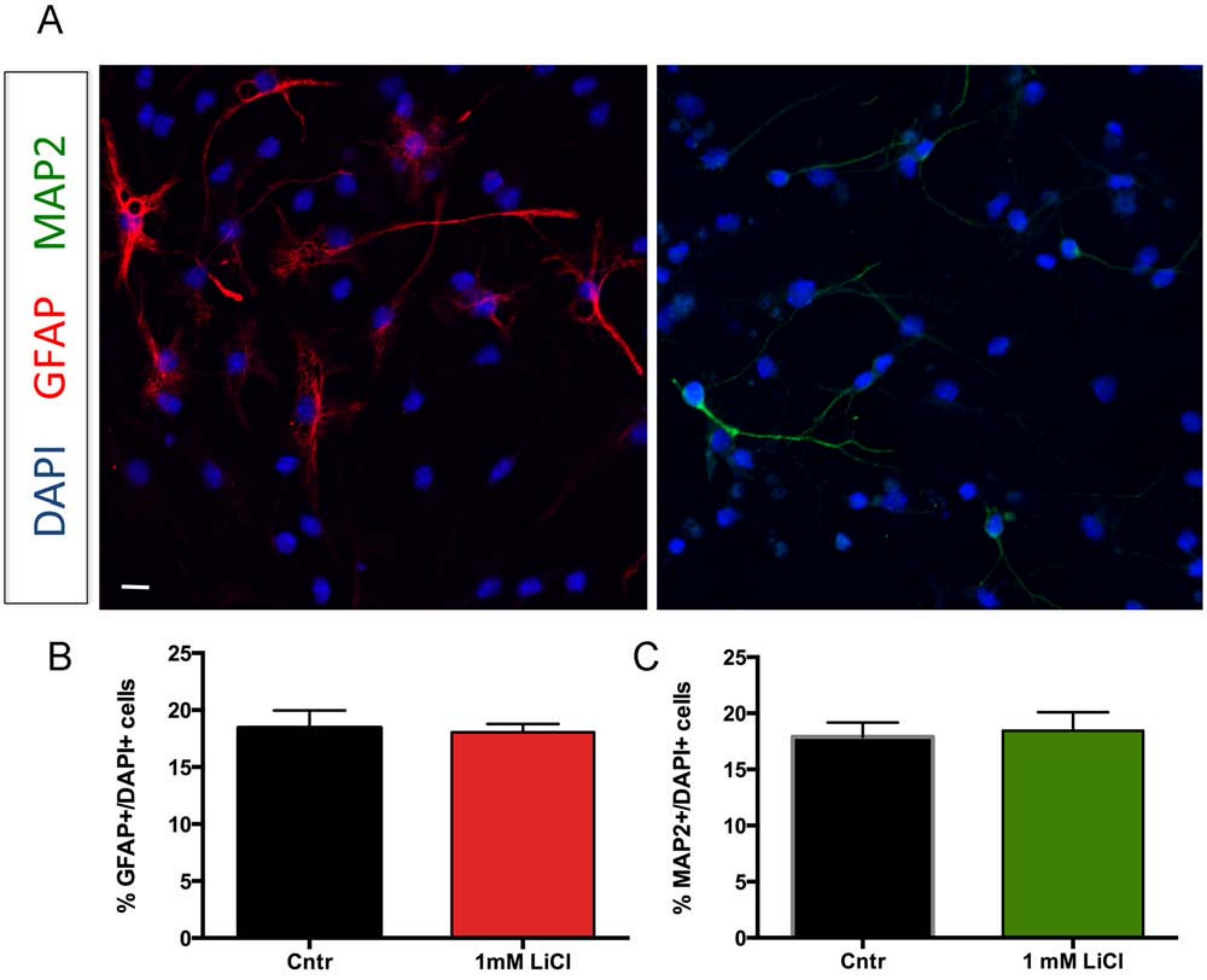

Supplementary Figure S1: Lithium did not affect the lineage commitment. A. Representative micrograph of the NSPCs after 7 days *in vitro*, showing mature neurons $MAP2^+$ (green), mature stellate astrocytes $GFAP^+$ (red) and nuclei positive for DAPI (blue). Magnification 20X, scale bar 10 µm. **B.** Quantification of the percentages of $GFAP^+$ cells showing no significant difference between control and lithium-treated NSPCs, $p = 0.8190$. **C.** Quantification of the percentages of $MAP2^+$ cells showing no significant difference between control and lithium-treated NSPCs, $p = 0.8002$. Data are presented as mean ± SEM, $n = 6$.

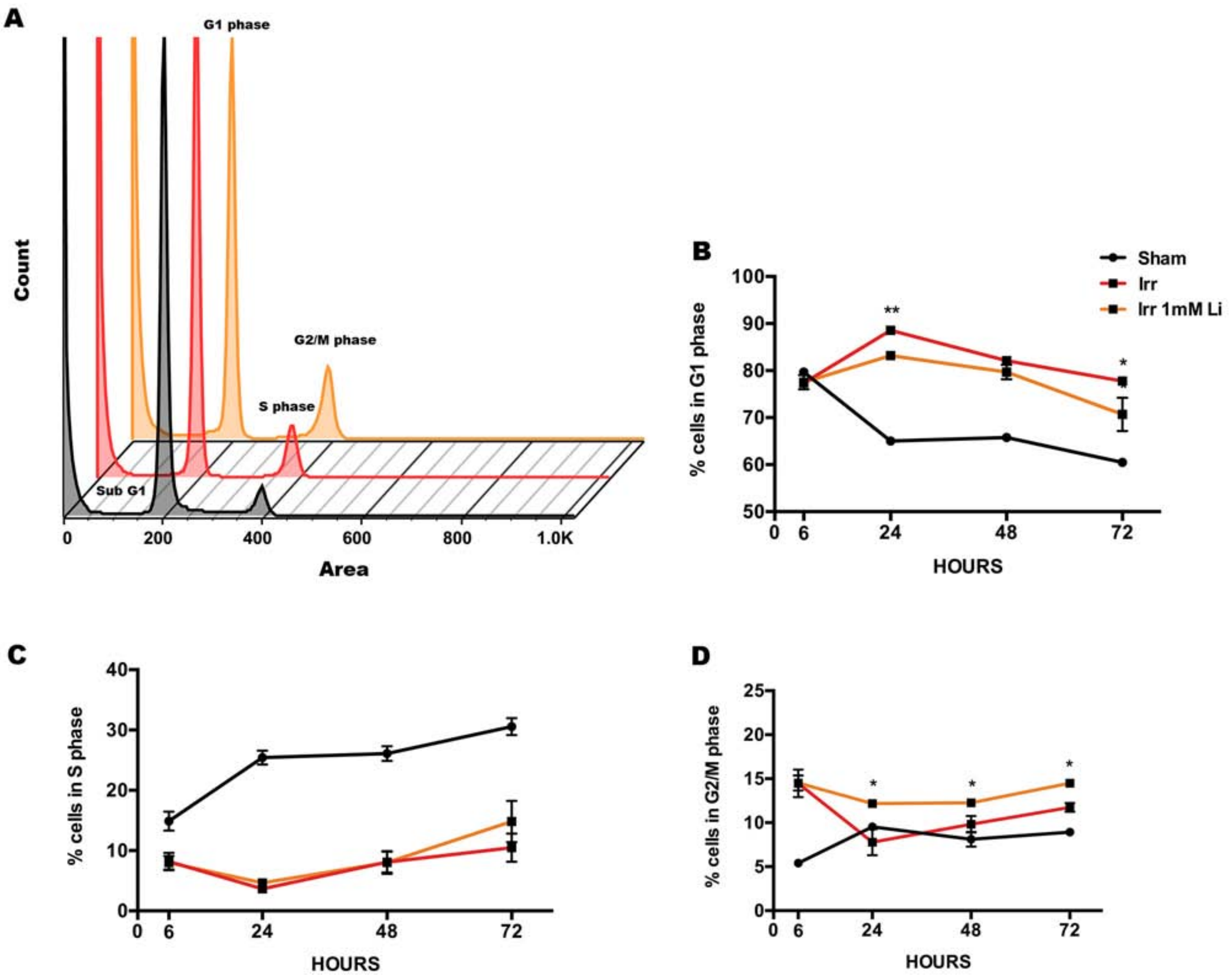

Supplementary Figure S2: Lithium at 1 mM dose induces an arrest in G2/M phase. A. Propidium iodide (PI) histograms showing the DNA distribution in G_1, S and G_2/M phases) in sham (black), irradiated (red) and irradiated+1 mM LiCl (orange). **B.** Quantification of the relative DNA distribution in G_1 phase at different times after irradiation showing that irradiation significantly arrests the cell cycle in G_1 ****$p_{24\ hours} < 0.0001$, *** $p_{48\ hours} = 0.0005$, * $p_{72\ hours} = 0.0308$. Lithium had a moderate effect in reducing the percentages of cells in G_1 phase at 24 and 72 hours times, **$p_{1\ mM\_24hours} = 0.0013$ and *$p_{1\ mM\_72hours} = 0.0324$. **C.** Time course of the percentages of cells in S phase showing no effect of 1 mM LiCl on proliferation but a significant reduction of proliferation due to irradiation at 24, 48 and 72 hours times *$p_{24hours} = 0.0299$, ****$p_{48hours} < 0.0001$, *$p_{72hours} = 0.0308$. **D.** Quantification of the percentages of cells in G_2/M phase over time after irradiation showing a significant stall of the cells in this phase following irradiation at 6 and 72 hours times **$p_{6hours} = 0.0016$, ***$p_{72hours} = 0.0002$. Treatment with 1 mM LiCl caused even higher accumulation of the cells in this cell cycle phase at 24, 48 and 72 hours times, *$p_{1\ mM\_24hours} = 0.0206$, *$p_{1\ mM\_48hours} = 0.0341$, *$p_{1\ mM\_72hours} = 0.0123$. Data are presented as mean ± SEM, *n* = 3–6.

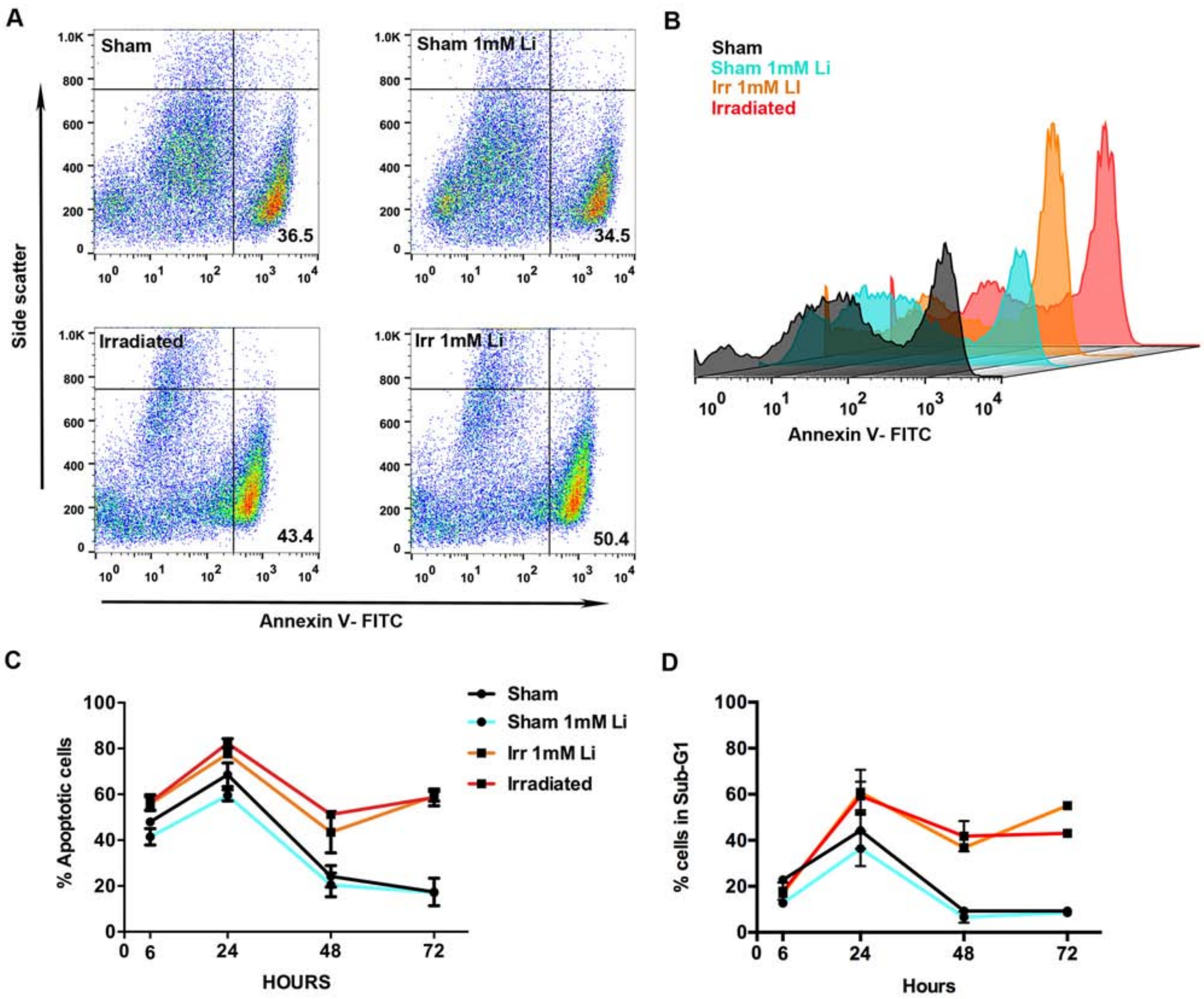

Supplementary Figure S3: Lithium did not halt irradiation-induced apoptosis in young neural stem cells. A. Representative scatter plot of the distribution of the cells stained with FITC for AnnexinV (x-axis) plotted against the side scatter (y-axis) analyzed by flow cytometer showing the percentages (%) of apoptotic cell in the lower right quadrant in each group: sham, sham+1 mM LiCl, irradiated and irradiated+1 mM LiCl. **B.** Representative picture illustrating the peaks of intensity of AnnexinV-FITC (x-axis) plotted against the count of the events (y-axis) in sham (black), sham+1 mM LiCl (light blue), irradiated (red) and irradiated+1 mM LiCl (orange). **C.** Quantification of the percentages of apoptotic cells, positive for annexin V, at different times showing that irradiation strongly induces apoptosis in this cell type at 6, 24, 48 and 72 hours times, $*p_{6hours} = 0.0371$, $***p_{24hours} = 0.0001$, $*p_{48hours} = 0.0219$, $***p_{72hours} = 0.0003$. Lithium at 1 mM did not rescue this cell type from apoptosis. **D.** Time course of the percentage of cells in sub-G_1. Irradiation displays an effect on cell death at 24, 48 and 72 hours, $*p_{24hours} = 0.0399$, $**p_{48hours} = 0.0016$, $****p_{72hours} < 0.0001$. Lithium at 1 mM did not rescue from apoptosis and it increased at 72 hours time point compared to irradiated only, $**p_{1\ mM\_72hours} = 0.0070$. Data are presented as mean ± SEM, n = 3–6.
